# Haptoglobin is an early indicator of survival after radiation-induced severe injury and bone marrow transplantation in mice

**DOI:** 10.1186/s13287-022-03162-x

**Published:** 2022-09-06

**Authors:** Shixiang Zhou, Yaqiong Li, Lexin He, Min Chen, Weihong Li, Ting Xiao, Jian Guan, Zhenhua Qi, Qi Wang, Siyuan Li, Pingkun Zhou, Zhidong Wang

**Affiliations:** 1 Department of Radiobiology, Beijing Key Laboratory for Radiobiology, Beijing Institute of Radiation Medicine, Beijing, 100850 China; 2 Department of Radiotherapy, Nanfang Hospital, Southern Medical University, Guangzhou, 510080 China; 3 Graduate Collaborative Training Base of Academy of Military Sciences, Hengyang Medical School, University of South China, Hengyang, 421001 Hunan China

**Keywords:** Haptoglobin (Hp), Acute radiation syndrome (ARS), Bone marrow transplantation, Biodosimeter

## Abstract

**Background:**

Hematopoietic stem cell transplantation (HSCT) is the main treatment for acute radiation sickness, especially after fatal radiation. The determination of HSCT for radiation patients is mainly based on radiation dose, hemogram and bone marrow injury severity. This study aims to explore a better biomarker of acute radiation injury from the perspective of systemic immune response.

**Methods:**

C57BL/6J female mice were exposed to total body irradiation (TBI) and partial body irradiation (PBI). Changes in haptoglobin (Hp) level in plasma were shown at different doses and time points after the exposure and treatment with amifostine or bone marrow transplantation. Student’s *t*-test/two tailed test were used in two groups. To decide the Hp levels as a predictor of the radiation dose in TBI and PBI, multiple linear regression analysis were performed. The ability of biomarkers to identify two groups of different samples was determined by the receiver operating characteristic (ROC) curve. The results were expressed as mean ± standard deviation (SD). Significance was set at *P* value < 0.05, and *P* value < 0.01 was set as highly significant. Survival distribution was determined by log-rank test.

**Results:**

In this study, we found that Hp was elevated dose-dependently in plasma in the early post-irradiation period and decreased on the second day, which can be used as a molecular indicator for early dose assessment. Moreover, we detected the second increase of Hp on the 3rd and 5th days after the lethal irradiation at 10 Gy, which was eliminated by amifostine, a radiation protection drug, while protected mice from death. Most importantly, bone marrow transplantation (BMT) on the 3rd and 5th day after 10 Gy radiation improved the 30-days survival rate, and effectively accelerated the regression of secondary increased Hp level.

**Conclusions:**

Our study suggests that Hp can be used not only as an early molecule marker of radiation injury, but also as an important indicator of bone marrow transplantation therapy for radiation injury, bringing new scientific discoveries in the diagnosis and treatment of acute radiation injury from the perspective of systemic immunity.

**Supplementary Information:**

The online version contains supplementary material available at 10.1186/s13287-022-03162-x.

## Introduction

With the continuous development of science and technology, nuclear energy is increasingly widely used in industry and other aspects. Large-scale nuclear accidents may occur at any time worldwide, causing a large number of casualties. If the estimation of radiation dose and classification of the injuries, especially the early identification of severely wounded, can be carried out for large-scale wounded in the early stage of the accident it will be an effectively guidance for medical personnel to formulate treatment schemes in time and minimize the casualties [1–4].

At present, there are two kinds of methods for radiation dose estimation: physical dose estimation and biological dose estimation. Due to the abruptness of the accidents, physical dosimeters are not always available to the exposed personnel, so the classification and dose assessment of mass casualties after nuclear accidents mainly relies on biological methods, including chromosome aberration analysis, lymphocyte count analysis and lymphocyte γ-H2AX analysis [5–9]. The chromosome aberration analysis is considered the “golden standard” for determining exposed dose due to its specificity and accuracy, but it also has disadvantages such as complex experimental method, high request for the operator, relatively narrow range from 0.1 to 5 Gy, and 48 h of cell cultivation before sample preparation and analysis [10, 11]. In recent years, chromosome analysis has achieved the automation of cell harvest, slice production and analysis, however, the 48-h cell culture is still required, and the dose upper limit remains 6 Gy [12]. Doses of ionizing radiation above 6 Gy can lead to significant hematopoietic function inhibition in human, which usually requires hematopoietic factor treatment and HSCT [13–15]. Hence a rapid and accurate assessment of severe injuries after an accident is of great importance to the application of HSCT and other therapeutic measures.

Ionizing radiation causes a dose-dependent decrease in peripheral blood lymphocyte number, and lymphocyte count analysis between 12 and 48 h after irradiation is used to classify patients with radiation injury. This method is fast and easy, but cannot be used within 12 h after exposure [16–18]. Therefore, the exploration of molecular biological radiation dosimeter is the key to implement early classification of mass casualties after nuclear accidents. Though current studies have reported potential molecular markers [19–21], the only molecular marker widely approved is peripheral blood lymphocyte γ-H2AX Foci analysis. γ-H2AX is a marker of DNA double-strand break. Obvious γ-H2AX foci emerges a few minutes after irradiation, peaks at about 1 h, and then gradually decreases. With the narrow detection window, its application is greatly limited [22–24].

Inflammation is a typical pathophysiological response to radiation and leads to tissue damage by exacerbating the direct effects of ionizing radiation [25]. The body appears an acute phase reaction early after exposed to exogenous stimulus such as ionizing radiation (several hours to one day), and some acute phase proteins show a rapid increase, usually begin to decline 24 h after exposure. Ionizing radiation can also cause apoptosis and necrosis in radiation-sensitive tissues such as hematopoietic, immune and intestinal cells [26, 27], especially after exposure to high-dose radiation, resulting in temporary changes or even loss of plasma membrane integrity. As a result, adenosine triphosphate (ATP), high mobility histone B1 (HMGB1), DNA, RNA and uric acid molecules that are normally expressed or found only in cells may leak out of damaged cells, forming damage associated molecular patterns (DAMPs) in the extracellular environment [28]. DAMPs can be perceived by neighboring tissue innate immune cells through some pattern-recognition receptors (PRRs) [29], and thus induce systemic inflammatory dysregulation in vivo, namely "cytokine storm", manifested by high levels of pro-inflammatory cytokines, such as IL-1β, IL-6, IL-18 and TNFα [26]. The cumulative systemic effects of inflammatory responses and platelet dysfunction can lead to multiple organ failure and death [27], which is an important factor in death caused by severe radiation. Hematopoietic stem cell/mesenchymal stem cells transplantation (HSCT/MSCs) is an important treatment for patients with severe injury caused by acute radiation and has shown to be effective in alleviating radiation-induced hematopoietic syndrome, intestinal syndrome, brain injury and lung injury [14, 15, 30–34]. At present, whether stem cell therapy is carried out depends mainly on dose assessment, bone marrow pathology analysis and comprehensive clinical manifestations. It is worth exploring whether biomarkers of early and severe injury can be detected during the radiation-induced acute response and DAMP process, for the sake of treatment and post-treatment evaluations of severe injuries.

Haptoglobin (Hp) is an acidic glycoprotein in the serum α 2-globulin component, which is widely found in the serum and other body fluids of humans and many mammals [35, 36]. As an acute stage protein, Hp is mainly synthesized and secreted by the liver and plays an important role in the process of anti-infection, repair of damaged tissues and stability of internal environment [37, 38]. Currently, Hp has been reported as a biomarker for the early diagnosis of lung cancer and the recovery of cervical cancer after radiotherapy [39, 40]. Although it was reported more than 20 years ago that the expression level of Hp significantly increased in the blood and bone marrow of C57BL/6 and CBA/Ca mice after 4-Gy whole-body γ ray irradiation, reflecting the sustained acute reaction [41], there are still few studies between Hp and ionizing radiation.

In this study, we confirmed plasma Hp as a sensitive and reliable radiation indicator in mice in a dose range relevant for triage after Total body irradiation (TBI) or local irradiation mouse models. Furthermore, the feasibility of Hp as a radiation biomarker was also verified in serum from patients with nasopharyngeal carcinoma after head and neck radiotherapy. Then, we found that Hp has a discriminative impact on nonlethal and lethal radiation, correlating with radiation dose as well as radioprotective agents. More importantly, Hp can also be used as an indicator after bone marrow stem cell transplantation in mice with severe radiation injury and one of the indicators of prognosis.

## Materials and methods

### Animals

C57BL/6J female mice (8-week-old) were obtained from SiBeiFu (Beijing) Biotechnology Co., Ltd and fed in the Beijing Institute of Radiology (Beijing, China). The mice were kept in a uniform cage under specific pathogen-free conditions, controlled temperature and humidity, under a 12/12 h light/dark cycle, for at least one week before the experimental treatment, weighing 19–21 g.

### Total body irradiation (TBI)

For time and dose response studies, mice were confined to a transparent plexiglass box and exposed to a single dose of 0.2, 0.5, 1, 2, 4, 6, 8, and 10 Gy radiation using a ^60^Co γ-ray at a dose rate of 68.6 cGy/min. 5 μl blood was added into 1 ml PBS for detecting dynamic changes of Hp by ELISA at − 3, 0.125, 0.25, 0.5, 1, 2, 3, 5, 7, 11 and 14 days after irradiation, and 20 μl blood was collected from the tail vein for blood counts at − 3, 0.125, 0.25, 0.5, 1, 2, 3, 5, 7, 11, 14, 20, 25 and 30 days. Mice were weighed, and their survival rate was observed until 30 days after irradiation.

For the study of in vivo Hp sources, mice were randomly allocated to experimental groups and left for 7 days to restore normal condition. Mice were placed in plexiglass transparent boxes to prevent movements and exposed to a single dose of 8 Gy radiation using ^60^Co γ-ray at a dose rate of 67.82 cGy/min. Mice were killed by decapitation at 1 day after irradiation and samples of large intestine, small intestine, lung, brain, liver, heart, spleen, adrenal gland, ovary, fat, thymus, kidney and bone marrow were dissected and stored at − 80 °C.

### Partial body irradiation (PBI)

Mice were anesthetized with sodium pentobarbital by intraperitoneal injection, and then exposed part of their bodies to a single dose of 10 Gy radiation using a ^60^Co source γ-ray at a dose rate of 68.55 cGy/min, using lead bricks as shields. Blood samples were collected from the tail vein for detecting dynamic changes of Hp by ELISA at − 3, 0.125, 0.25, 0.5, 1, 2, 3, 5, 7, 11 and 14 days after irradiation, and at − 3, 0.125, 0.25, 0.5, 1, 2, 3, 5, 7, 11, 14, 20, 25 and 30 days for blood cell count. Mice were weighed after irradiation and their survival rate were observed until day 30.

### Amifostine treatment

Mice were treated with 150 mg/kg amifostine by intraperitoneal injection 0.5 h before 10 Gy irradiation and then exposed to a single dose of 10 Gy using ^60^Co γ-ray, the radiation was carried out at a dose rate of 69.45 cGy/min. Blood samples were collected from the tail vein for detecting dynamic changes of Hp by ELISA at − 3, 1, 2, 3, 5, 7, 11 and 14 days after irradiation, and at − 3, 1, 2, 3, 5, 7, 11, 14, 20, 25 and 30 days for blood cell count. Mice were weighed after radiation and their survival rate was observed until day 30.

### HE staining and bone marrow nucleated cell count of mouse femur

Femur wsd dissected from control and radiated mice immediately after euthanasia at 0.25, 3 and 7 days after a single dose of 6.5 Gy radiation using ^60^Co γ-ray at a dose rate of 60.91 cGy/min, fixed in 4% paraformaldehyde for 24 h. For histological analysis, the femur was dehydrated, paraffin-embedded. Five-micrometer thick sections were cut and mounted on glass slides. After deparaffinization, the sections were processed for Hematoxylin and Eeosin (H&E) staining analysis. The images of femoral bone marrow were acquired with a microscope (Y-TV55, Nikon, Japan) and Nikon digital imaging system (DS-Ri2, Nikon, Japan).

Bone marrow nucleated cells in the same size fields selected randomly from images of femoral bone marrow were counted, and Student’s *t*-test was used to evaluate the significant difference.

### Bone marrow transplantation assay

Whole bone marrow cells were flushed out from the femurs of donator mice with D-Hank’s buffer, and filtered using 40-μl Cell Strainer (15–1040, BIOLOGIX, Jinan, China). We selected the number of transplanted cells according to the literature [42]. In order to improve the survival rate, we doubled the number of transplanted cells on the 1st, 3rd day after irradiation. Bone marrow cells in 300 μl D-Hank’s with 10% fetal bovine serum (FBS) were intravenously injected into recipient through the tail vein at 1 day (10 Gy-1d-T, 8 × 10^6^ cells), 3 days (10 Gy-3d-T-1, 8 × 10^6^ cells), (10 Gy-3d-T-2, 1.6 × 10^7^ cells)), and 5 days (10 Gy-5d-T-1, 8 × 10^6^ cells), (10 Gy-5d-T-2, 1.6 × 10^7^ cells), (10 Gy-5d-T-3, 2.4 × 10^7^ cells) after 10 Gy radiation using a ^60^Co γ-ray at a dose rate of 66.87 cGy/min, survival rate of mice was observed everyday after bone marrow transplantation. Blood samples were collected from the tail vein for detecting dynamic changes of Hp by ELISA at − 3, 1, 2, 3, 5, 7, 11 and 14 days after irradiation, and at − 3, 1, 2, 3, 5, 7, 11, 14, 20, 25 and 30 days for blood cell count. Mice were weighed after radiation and their survival rate was observed until day 30.

### Patients serum

Human serum was collected from 18 patients with nasopharyngeal carcinoma (NPC). All patients have signed written informed consents in Southern Medical University of China. All specimens were obtained from the clinical research initiation project of Southern Medical University funded by the high level university construction fund of Guangdong Provincial Department of Education (LC2016 PY015), which could be found at https://www.clinicaltrials.gov/. The photon beam emitted by 6 MV linear accelerator was used on the nasopharynx and its adjacent areas, such as the posterior nasal fossa, some of the paranasal sinuses, parapharyngeal space and skull base. The total target dose was 70 Gy in nasopharynx and 63 Gy in the neck. Blood samples were collected 3–7 days before radiotherapy and 1–4 days after radiotherapy. The serum was centrifuged at 3000 rpm/min at 4 °C for 10 min and stored at − 80 °C.

### Enzyme linked immunosorbent assay (ELISA)

The concentration of plasma Hp in mice was quantified using mouse Hp ELISA kits (Mouse Hp: EK1698, Boster, Wuhan, China); The concentration of Hp in the blood serum of human was quantified using human Hp ELISA kits (Human Hp: KE00148, Proteintech, USA). 100 μl of diluted plasma sample or standard sample of different concentration was added to microwells which were pre-coated by Hp antibody, and incubated for 1.5 h at 37 °C. Then the liquid in the microwells was discarded, 100 μl diluted (1:100) anti-mouse Hp antibodies was added to each well and incubated at 37 °C for 1 h. After washings with washing buffer, 100 μl of diluted (1:100) horseradish peroxidase was added to each well and incubated at 37 °C for 30 min. After washing five times, 90 μl of tetramethylbenzidine (TMB) was added to each well and incubated at 37 °C for 20 min before adding stop solution. Then the concentration of Hp was measured at 450 nm.

### RNA isolation and real-time PCR array analysis

Dissected tissues were cut and grinded in 1 ml TRIzol (T9424, Sigma, USA). Total RNA was isolated according to instructions. The cDNA was synthesized using PrimeScript RT reagent kit (RR047A, Takara, Japan) according to instructions. Real-Time PCR was performed using iTaq Universal SYBR Green Supermix (172–5124, BioRad, USA) on BioRad CFX96. Primers are listed as follows: Hp (sense 5′-AAAAACCTCTTCCTGAACCAC-3′, antisense 5′-AACGACCTT CTCAATCTCCAC -3′), β-Actin (sense 5′-AAGATCAAGATCATTGCTCCTCC-3′, antisense 5′-GACTCATCGTACT CCTGCTTGC-3′).

### Statistical analysis

The studies were blinded during data collection and quantification. Data in figure panels reflect several independent experiments performed on different days. Sample sizes can be found within results and/or figure legends, and individual data points are shown for each quantification. No statistical methods were used to predetermine sample size in other experiments. Student’s *t*-test/two tailed test were used in two groups. To decide the Hp levels as a predictor of the radiation dose in TBI and PBI, Multiple Linear Regression Analysis was performed. The ability of biomarkers to identify two groups of different samples were determined by the receiver operating characteristic (ROC) curve. The results were expressed as mean ± standard deviation (SD). Significance was set at *P* value < 0.05 and *P* value < 0.01 was set as highly significant. Survival distribution was determined by log-rank test.

## Results

### Plasma Hp is a sensitive and reliable radiation indicator in mice in a dose range relevant for triage after TBI

**Fig. 1:**
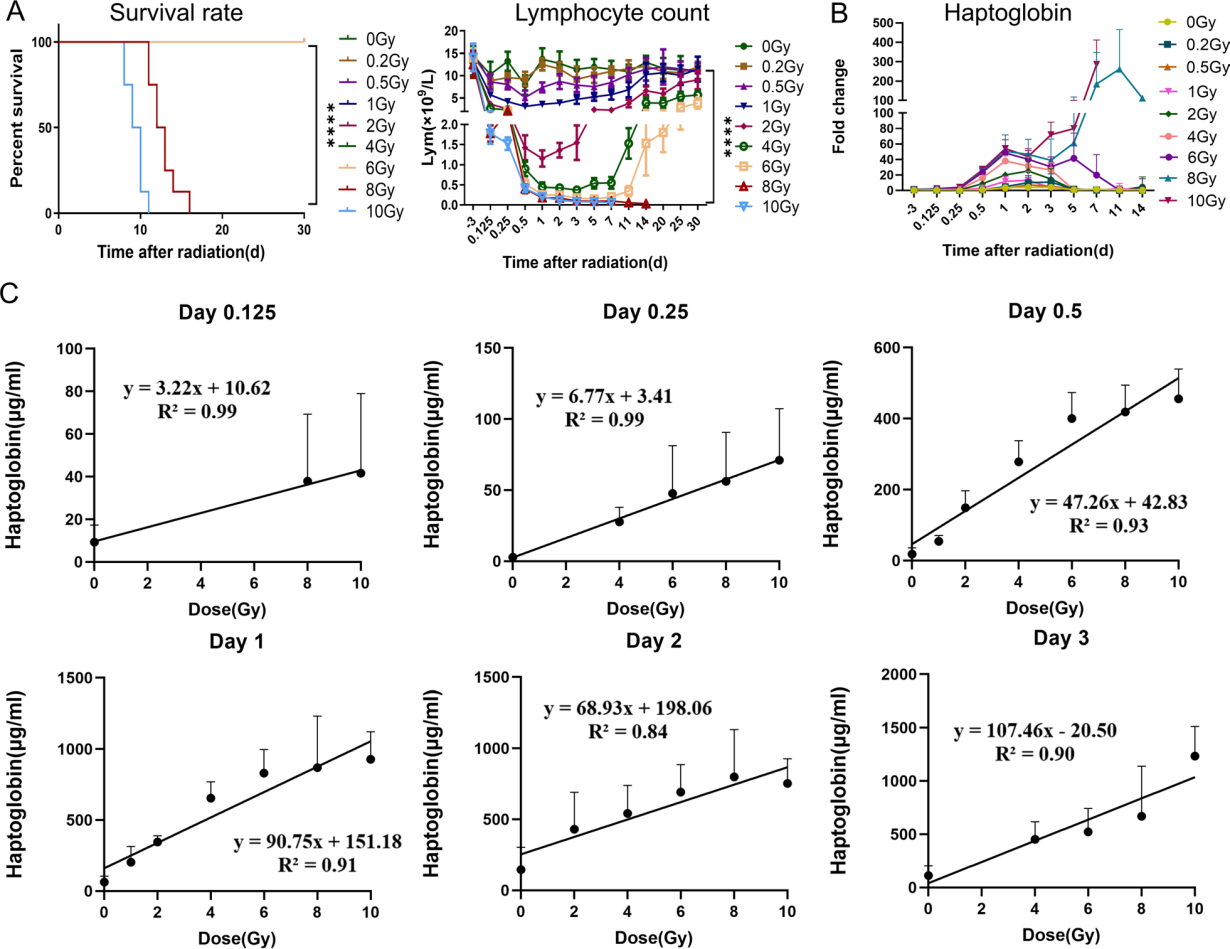
Plasma Hp dose-dependent increases in mice after exposure to different doses of TBI. **A** Survival rate, and Lymphocyte count. The *P* value of survival rate determined by log-rank (Mantel-Cox) test. Data are mean ± SD of *n* = 8 mice per group, statistical significance by Student’s *t*-test. *Hp* haptoglobin, *TBI* total body irradiation. ****, *P* < 0.0001. ***, *P* < 0.001. *, *P* < 0.05. **B** Fold change in Hp from plasma of animals in **A** at different time after TBI. Data are mean ± SD of *n* = 8 mice per group, statistical significance by Student’s *t*-test. *Hp* haptoglobin, *TBI* total body irradiation. Additional file 1: Table S1 shows fold change and *P* value of the data. **C** The correlation between Hp concentration and radiation dose at 3 h, 6 h, 12 h, 1 day, 2 days and 3 days after irradiation

To identify whether changes in plasma Hp levels after TBI were correlated with radiation doses, we detected plasma Hp in C57BL/6J mice exposed to 0 (control), 0.2, 0.5, 1, 2, 4, 6, 8, and 10 Gy of TBI at different time points (− 3, 0.125, 0.25, 0.5, 1, 2, 3, 5, 7, 11 and 14 days) after radiation (*n* = 8) by ELISA. At the same time, we established mouse models with different severity of radiation injury, and observed the survival rate, peripheral blood hemogram and body weight of mice after TBI (Fig. 1A, Additional file 1: Fig. S1). It was found that all mice died after 8 Gy and 10 Gy irradiation, and there was no death in other dose groups. At different time points, compared with the control group, lymphocytes (Lym), leukocytes (WBC), hemoglobin (HGB) and platelets (PLT) in the irradiation group decreased in varying degrees. The lymphocytes of 0.2–6 Gy irradiated mice almost completely recovered 30 days after irradiation, while the lymphocytes of 8 Gy and 10 Gy irradiated groups decreased from 3 h after irradiation and gradually decreased until death. It is shown that the early period of ionizing radiation, the concentration of Hp increased in a dose-dependent manner (Fig. 1B and Additional file 1: Table S1). 0.2 Gy and 0.5 Gy groups increased at 2d and 3d after irradiation, but there was no significant difference (Fig. 1B). In the 1 Gy and 2 Gy irradiation groups, it increased at 12 h, peaked on day 2 and returned to normal on day 3. 4 Gy group began to rise at 6 h, peaked on the first day, decreased on the second day, and returned to normal on the fifth day. The concentration of Hp increased at 6 h after sublethal dose of 6 Gy radiation, reached the peak on the first day, decreased on the second day, and returned to normal on the 11th day. Three hours after lethal dose (8 Gy and 10 Gy) irradiation, the concentration of Hp increased twice, and there was a significant difference compared with the normal control group. After reaching a high level on 1 day, it decreased slightly on 2 days, picked up again on 3 or 5 days, and continued to increase until death, while the nonlethal group gradually returned to normal after reaching the peak. Interestingly, the time of death was inversely proportional to the concentration of Hp in the 8 Gy and 10 Gy irradiation groups, and the time of death was later in the groups with lower Hp levels (Additional file 1: Table S1). These data suggest that Hp responded to all doses and times, and decreased slightly at day 2, then increased continuously until death after lethal dose TBI (Additional file 1: Table S1).

As described above, our study identified a radiation dose-dependent increase in plasma Hp, indicating its potential use as a radiation biodosimetry. To explore whether plasma Hp level can be used as a predictor of radiation dose in mice, we analyzed the correlation between Hp concentration and radiation dose at 3 h, 6 h, 12 h, 1 day, 2 days and 3 days after irradiation by linear regression. The correlation coefficient was above 0.9 except for day 2, and the highest was 0.99 at 6 h and 12 h. It is strongly suggested that Hp may be a potential indicator for predicting ionizing radiation exposure (Fig. 1C).

### The levels of Hp increased in mouse plasma and human serum after PBI

**Fig. 2:**
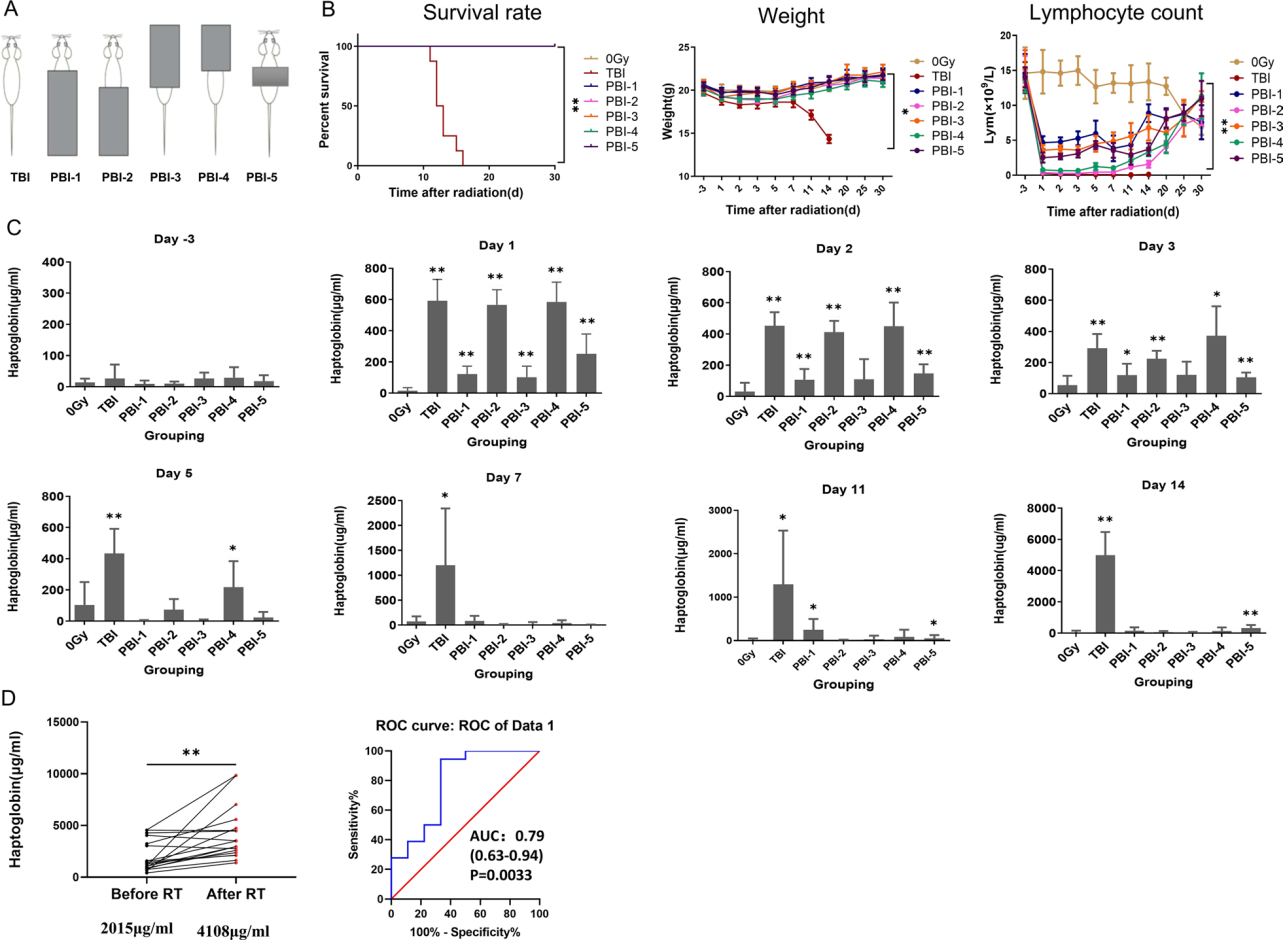
Increased Hp levels in mice and human after PBI. **A** The liver of C57BL/6J mice was covered in PBI-1 and PBI-3 and in the corresponding groups PBI-2 and PBI-4 the liver was exposed. **B** Survival rate, Body weight, and Lymphocyte count. The *P* value of survival rate determined by log-rank (Mantel-Cox) test. Data are mean ± SD of *n* = 8 mice per group, statistical significance by Student’s *t*-test. Hp, haptoglobin; PBI, partial body irradiation. *, *P* < 0.05, and **, *P* < 0.01 in the TBI irradiated mice compared with the control mice. **C** Hp was measured using ELISA in TBI and PBI group at 0.125, 0.25, 0.5, 1, 2, 3, 5, 7, 11 and 14 days after 10 Gy radiation. Data are mean ± SD of *n* = 8 mice per group, statistical significance by Student’s *t*-test. **, *P* < 0.01, *, *P* < 0.05 in the radiated mice compared with the control mice. **D** Changes in serum Hp in patients with nasopharyngeal carcinoma after radiotherapy. The scatter plot shows the expression of Hp concentrations in 18 patients with nasopharyngeal carcinoma before and after radiotherapy (**, *P* < 0.01). The ROC curve shows that Hp was used as a biomarker to predict radiation exposure in patients with nasopharyngeal carcinoma

To confirm the source of Hp due to irradiation, Hp mRNA expression in various tissues of C57BL/6J mice after TBI was measured. We found that liver is the main source of Hp after 8 Gy TBI (Additional file 1: Fig. S2A), and to further test the function of liver in Hp synthesis and secretion, five kinds of PBI modes were used: exposing to radiation the head and the chest (PBI-1), hiding the head and the chest (PBI-4), hiding the hypogastrium and hind legs (PBI-2), exposing to radiation the hypogastrium and hind legs (PBI-3) and hiding the liver (PBI-5) (Fig. 2A). The survival rate, body weight and peripheral blood hemogram of local irradiation mouse within 30 days were observed (Fig. 2B, Additional file 1: Fig. S2B). All mice in the TBI group died, and there was no death in other groups. The body weight of the TBI group decreased continuously, and there was no significant decrease in other groups. Lymphocytes began to decrease significantly on day 1, which was negatively correlated with the irradiation area; the number of leukocytes was similar to that of lymphocytes; Platelet count and hemoglobin decreased later. In addition, the plasma Hp concentrations of C57BL/6J mice exposed to 0 Gy (control group) and 10 Gy PBI at different time points (− 3, 1, 2, 3, 5, 7, 11 and 14 days) after radiation (*n* = 8 animals per group) were analyzed by ELISA. It was found that after PBI, although the liver was shielded as the main tissue producing Hp, the plasma Hp of mice increased regardless of shielding or exposing the liver, and was positively correlated with the irradiation area (Fig. 2C). It fully shows that the change of Hp can reflect the damage state of the whole body and can be used as a marker of local irradiation in mice.

As shown above, plasma Hp is a sensitive and robust radiation indicator in a dose range relevant for triage after TBI and PBI in a mouse model. We then investigated whether Hp can be used as an indicator in human. Serum Hp concentrations were estimated after head and neck radiotherapy in patients with NPC. The serum of 18 NPC patients was tested in this study (Additional file 1: Table S2). It showed a higher serum Hp concentrations after radiotherapy (Fig. 2D), and the mean concentration after radiotherapy (4108 μg/ml) was significantly higher than that before treatment (2015 μg/ml). We used receiver operating characteristic curve (ROC curve) to analyze whether Hp in serum of 18 NPC patients can be used as a potential diagnostic biomarker for radiotherapy patients. Results are shown in Fig. 2D, the area under the ROC curve (AUC) was 0.79, *P* = 0.0033. Hp can be used to distinguish the serum of patients before and after radiotherapy.

### Hp distinguishing impact of nonlethal and lethal radiation, not only radiation dose, correlate with survival

**Fig. 3:**
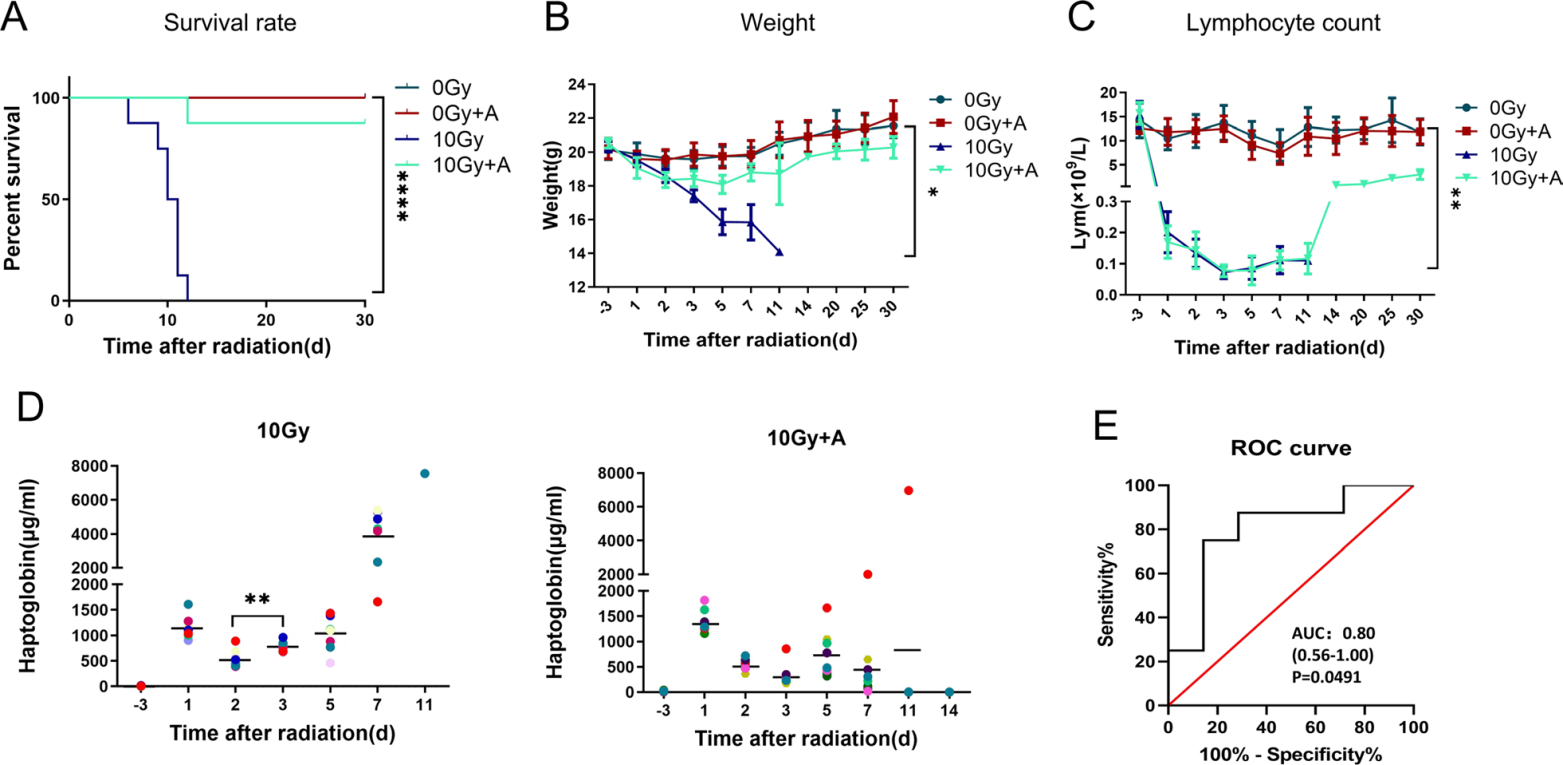
Plasma Hp levels discriminating lethal radiation. **A** Kaplan–Meier survival curves of mice (Survival rate). *P* value determined by log-rank (Mantel-Cox) test. **B** Weight and **C** Lymphocyte count. Data are mean ± SD of *n* = 8 mice per group, statistical significance by Student’s *t*-test. *, *P* < 0.05, **, *P* < 0.01, and ****, *P* < 0.0001 in the TBI mice compared with the control mice. **D** Hp was measured using ELISA in 10 Gy, 10 Gy + A, irradiated female C57BL/6J mice at − 3, 1, 2, 3, 5, 7, 11 and 14 days post-irradiation. Error bars indicate ± 1 SD for each radiation exposure group. *n* = 8 per group (*n* = 1 at 11 days after 10 Gy). **, *P* < 0.01. *A* amifostine, *Hp* haptoglobin, *TBI* total body irradiation. **E** The ROC curve shows that Hp was used as a biomarker to distinguish between 10 and 10 Gy + A. *n* = 8 mice per group

As shown Fig. 1, Hp concentrations in plasma of mice increased again 3 days after lethal dose irradiation. To determine whether the change of plasma Hp correlated with acute radiation inducing fatal injury, we injected a radioprotective agent amifostine, which is known to prolong survival in mice and in humans by reducing radiation-related cytotoxicity, into mice intraperitoneally half an hour before 10 Gy TBI. Then, we observed whether amifostine could relieve the secondary increase of Hp in plasma while improving the survival rate of lethal irradiated mice. Results as shown in Fig. 3A, all mice exposed to 10 Gy lethal radiation died within 12 days, whereas only one of mice in the amifostine pretreatment group died. The analysis of body weight (Fig. 3B) and hemogram (Fig. 3C and Additional file 1: Fig. S3A) of irradiated mice showed that amifostine pretreatment could reduce the weight loss of irradiated mice and promote recovery, but did not change the decline of radiation-sensitive indicators such as lymphocytes. The dynamic Hp concentration and corresponding survival time of each mouse in 10 Gy group and 10 Gy + A are shown in Fig. 3D and Additional file 1: Table S3, in which each row expresses the Hp level of continuous blood sample from a single mouse and corresponding survival time. Plasma Hp in 10 Gy group increased to a higher level on the first day after irradiation, decreased slightly on the second day, and then continued to increase again until death. However, the plasma Hp level of mice pretreated with amifostine before 10 Gy irradiation began to decrease after reaching the highest level on the first day after irradiation and all 7 mice survived within 30 days after irradiation without secondary increase of Hp and continued to decline to the normal level, one mouse died 12 days after irradiation, and the change law of Hp level in this mouse was consistent with that in the simple 10 Gy irradiation group, with an obvious secondary increase. These results further suggest that the secondary increase of plasma Hp in mice 3–5 days after irradiation can be used as a useful indicator to predict the survival of mice. ROC curve results showed that mouse plasma Hp could be used to distinguish nonlethal and lethal radiation (Fig. 3E, area under curve (AUC) was 0.80, *P* = 0.0491).

### Hp is a molecular indicator for timing selection and therapeutic effect evaluation of bone marrow transplantation in mice with severe radiation injury

**Fig. 4:**
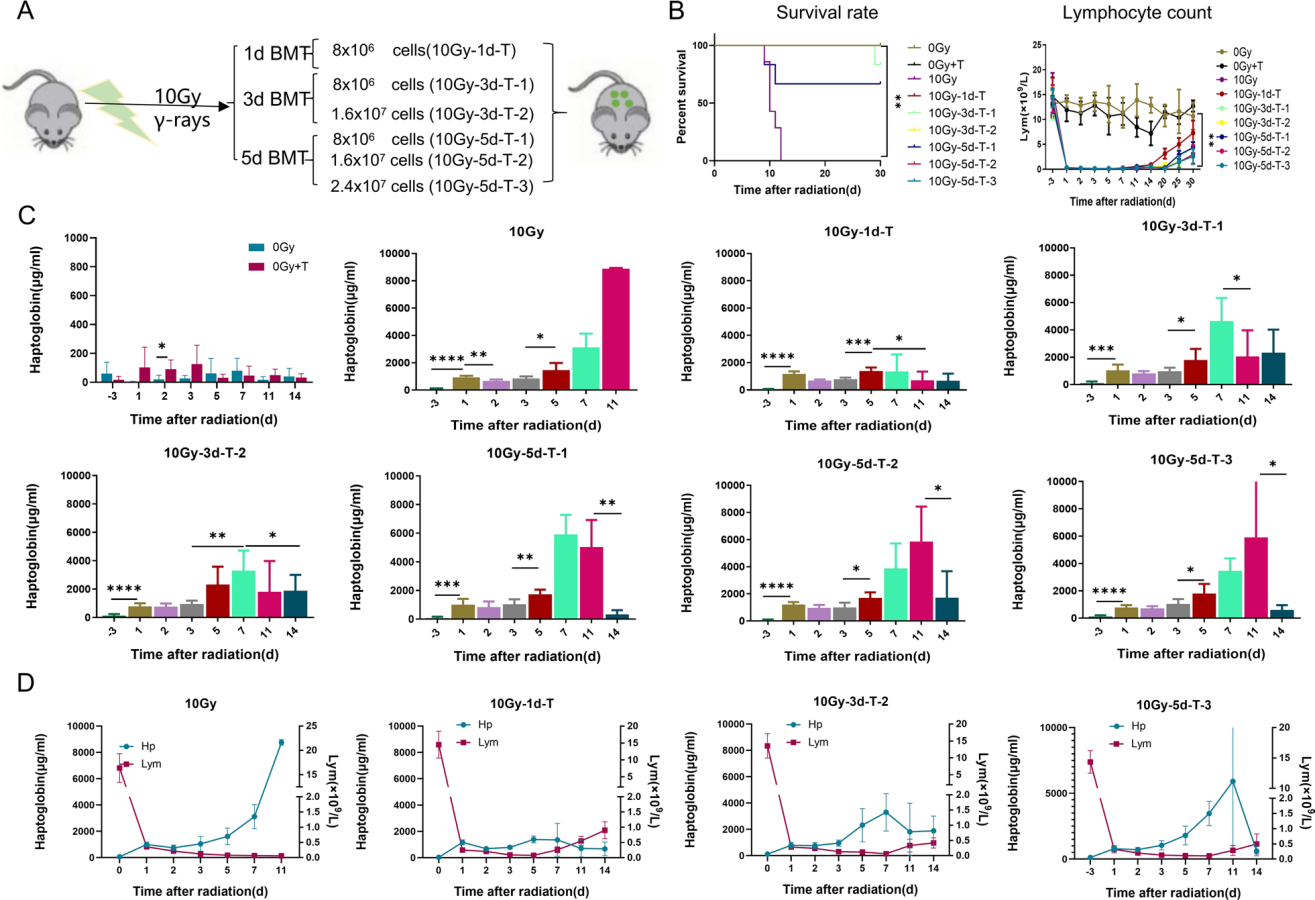
Changes in plasma Hp concentrations after BMT in mice with severe radiation injury. **A** Schematic to describe transplantation of whole bone marrow (WBM) into lethally irradiated recipients. BMT, bone marrow transplantation. **B** Survival rate, Lymphocyte count in irradiated mice. The *P* value of survival rate determined by log-rank (Mantel-Cox) test. **C** Hp was measured using ELISA in 0 Gy, 0 Gy + T, 10 Gy, 10 Gy-1d-T, 10 Gy-3d-T-1, 10 Gy-3d-T-2, 10 Gy-5d-T-1, 10 Gy-5d-T-2, 10 Gy-5d-T-3, irradiated female C57BL/6J mice at different time points. Data are mean ± SD of *n* = 6 mice per group except 10 Gy-3d-T-2 (*n* = 5), statistical significance by Student’s *t*-test. *, *P* < 0.05; **, *P* < 0.01; ****, *P* < 0.0001. Additional file 1: Table S4 shows the *P* value of Hp concentrations after comparing with the previous time point. **D** Comparison of lymphocytes and Hp in 10 Gy, 10 Gy-1d-T, 10 Gy-3d-T-2, 10 Gy-5d-T-3, irradiated female C57BL/6J mice at different time points. Data are mean ± SD of *n* = 6 mice per group except 10 Gy-3d-T-2 (*n* = 5), statistical significance by Student’s *t*-test. *, *P* < 0.05; **, *P* < 0.01. *T* transplantation, *A* amifostine, *Hp* haptoglobin

As the results of HE staining and the count of nucleated cells in bone marrow of mice in the control group and the 6.5 Gy irradiation group were shown in Additional file 1: Fig. S4, the dose of ionizing radiation above 6 Gy would cause bone marrow injury. Stem cell transplantation is the most important clinical treatment. Generally, the earlier the transplantation, the more significant the curative effect. However, existing technologies used to evaluate the extent of radiation have notable limitations, it is impossible to accurately judge whether patients need transplantation. Based on the fact that plasma Hp concentration can effectively identify lethal radiation injury, we hope to further explore whether plasma Hp concentration can be used as an indicator of the necessity of transplantation treatment. Different numbers of whole bone marrow cells (Fig. 4A) were reinfused into the tail vein of 10 Gy irradiated mice on the 1st, 3rd and 5th day after irradiation. Then, we analyzed the survival rate, body weight and peripheral hemogram of mice in each group within 30 days after irradiation. Results as shown in Fig. 4B and Additional file 1: Fig. S5, all mice in 10 Gy irradiation group died within 12 days after irradiation. Bone marrow transplantation of all BMT-treated groups effectively improved the survival rate of mice and promoted the recovery of body weight, hemogram and other indicators. The survival rate of 10 Gy-1d-T was 100%, and a few mice died in 10 Gy-3d-T-1 and 10 Gy-5d-T-1 groups, with survival rates of 83% and 67%, respectively. When the number of transplanted cells was increased, the survival rate of mice in 10 Gy-3d-T-2, 10 Gy-5d-T-2 and 10 Gy-5d-T-3 groups increased to 100%. These results suggest that selecting the time of bone marrow transplantation according to the changes of plasma Hp in irradiated mice can effectively treat lethal dose irradiated mice (Fig. 4C and Additional file 1: Table S4).

Bone marrow transplantation can effectively accelerate the recovery of weight and hemogram of mice after irradiation. At the same time, the recovery of lymphocyte and other indicators is also an important indicator for the success of bone marrow transplantation and the recovery of radiation injury. Next, we analyzed whether the level of Hp in plasma can be used to evaluate the therapeutic effect of bone marrow transplantation in irradiated mice. The results showed that bone marrow transplantation after irradiation could effectively promote the recovery of Hp level after 10 Gy irradiation. The earlier the transplantation time and the more the number of transplanted cells, the faster the recovery of Hp to normal level, which was similar to the recovery time of peripheral blood lymphocytes (Fig. 4D), suggesting that Hp can be used as an auxiliary indicator of the therapeutic effect of bone marrow transplantation.

## Discussion

Nuclear- terrorist attacks and accidental explosions of civil nuclear facilities can cause a large number of nuclear radiation casualties in a short time. The intensity and latency of acute radiation syndrome (ARS) and late effects can vary depending on the absorbed dose and exposed area of the body. Rapid and accurate dose estimation and injury classification at the early stage of nuclear accident, especially accurate identification of the critically injured, can effectively guide the formulation of personalized medical schemes and allocation of medical resources to minimize casualties, making it the top priority of medical rescue [43, 44].

Molecular biomarkers are of great significance for the identification, early diagnosis and prevention, monitor during treatment and after-treatment evaluation of diseases, and have been widely applied in the clinical diagnosis and after-treatment evaluation of various diseases. With the rapid development of various omics techniques, the study of molecular markers specifically for radiation injury has been widely carried out. Potential molecular markers reported by current studies include small metabolite molecules, non-coding RNA, cytokines, chemokines and other proteins [19–21, 27]. Citrulline, a nitrogen end-product of intestinal glutamine metabolism, has been identified as a potential circulating biomarker of radiation-induced gastrointestinal injury and epithelial cell loss, and its plasma concentration is negatively correlated with gastrointestinal tissue injury [45–47]. However, as a specific marker of intestinal injury caused by ionizing radiation, citrulline could not reflect the severity of systemic injury in the exposed subjects, and it changed significantly only exposed to radiation more than 6.5 Gy. miR-RAD (miR-150-5p/miR-23a-3p) as a sensitive and robust radiation biodosimeter in mice in a dose range relevant for triage after radiological events, however, resolvability decreased at doses beyond 4 Gy [48]. γ-H2AX was found to be a reliable and highly sensitive marker of radiation-induced DNA double-strand break [49, 50]. γ-H2AX foci forms after exposed a few minutes to DNA damage factors such as ionizing radiation, rapidly to the peak value within 1 to 3 h after exposure and then decreasing gradually, leaving no obvious plateau period [50, 51]. Therefore, γ-H2AX foci analysis has advantages of high sensitivity, as well as disadvantages like short detection window. In this study, we found that the plasma Hp level of mice increased rapidly with a significant dose dependence after 3 h of TBI more than 8 Gy, 6 h after irradiation more than 4 Gy, and 12 h after irradiation more than 1 Gy, reaching the peak level at 24–48 h. Subsequent local irradiation experiments confirmed that plasma Hp of irradiated mice mainly came from liver, heart and thymus tissues, and was closely related to the severity of radiation damage. Clinical samples from patients with nasopharyngeal carcinoma confirmed that Hp is also radio-sensitive in humans. Therefore, the change of plasma Hp concentration in mice after exposure has a strong sensitivity and time-dose effect, which can be used for the classification of patients in the early stage of accident. Furthermore, Hp, as plasma protein, is more easily to be used for rapid detection at accident site, and the detection window is wider, which can be more valuable in medical rescue in large accidents.

Mang et al. [52] found that 5 Gy radiation did not increase urine interleukin-18 (IL-18). High-dose TBI significantly increased urine IL-18 at day 1 to day 5 in a bell-shaped time course, reaching a peak of 5- to 10-fold of control levels on day 3 after 6.5 Gy and 8.5 Gy, respectively. It suggests that urine IL-18 can be used as a biomarker to assess the risk of radiation-induced death. In addition, Kiang et al. [53] also found that ionizing radiation increased the serum circulating IL-18 concentration of mice continuously. Meanwhile, radiation-induced G-CSF levels increased dose- and time-dependently, peaking at 8 h post-radiation and then decreasing, and the second increase was observed in mice irradiated with sublethal and lethal doses (≥ 6 Gy). This suggests that G-CSF together with IL-18 can be used as a biomarker to validate the assessment of radiation exposure and severity of tissue injure, and to estimate the risk of death after radiation injury. Other molecular indicators of radiation injury, such as FLT-3 ligand, lymphocyte γ-H2AX and citrulline, cannot be used for early prediction of radiation-induced death [27, 47, 54, 55].

In our study, the plasma Hp dropped to normal after the dose-dependently reached its highest point, and the recovery time extends with the increase in dosage, irradiated by dose below 6 Gy. In the mice exposed to 8 Gy and 10 Gy, Hp increased at 3 h after radiation, peaked at 1 day and began to fall, followed by a second rise on the 3rd to 5th day, and then continued to increase until death. We also found that amifostine can effectively inhibit the secondary elevation in plasma Hp in mice irradiated at 10 Gy. In the only one mouse that died after amifostine pretreatment, the change in plasma Hp concentrations was consistent with that in the untreated group, showing a second increase and continued to increase until death. However, amifostine had no inhibitory effect on the decrease in lymphocyte counts. Other hematologic indicators (WBC, HGB, PLT) were also not affected by amifostine. These results suggest that second increase in plasma Hp concentrations is closely associated with mice death. In addition, the dynamic changes in plasma Hp concentrations are closely related to radiation effect, rather than determined by exposure dose. Plasma Hp has more application value as it can reflect the severity of injure induced by ionizing radiation directly and effectively avoid individual variation during injuries classification after accident.

Infection and hemorrhage subsequent to bone marrow hematopoietic failure are the major causes of death from ARS. 2 Gy TBI can cause significant decrease in the lymphocytes count, which usually requires medical intervention, while irradiation at 6 Gy or above can cause severe hematopoietic function inhibition, which should be treated by hematopoietic factor application or HSCT [13–15, 34, 56]. However, the diagnosis and treatment of ARS largely depend on the estimation of irradiation dose. Due to the limitations of current radiation dose estimation techniques, it is difficult to accurately determine whether patients need transplantation, and the optimal time for HSCT is often lost or Medical resources are wasted due to excessive medical treatment [57]. As plasma Hp concentrations can effectively identify lethal radiation injury, we further explore whether plasma Hp concentrations can be used as an indicator of the need for transplant therapy. Bone marrow cells were transplanted into mice at different times after lethal irradiation. Analysis of survival rate of mice within 30 days after irradiation showed that bone marrow transplantation could improve the survival rate of mice after 10 Gy irradiation. The detection of the weight and hemogram of mice showed that the recovery of Weight and lymphocyte in mice after bone marrow transplantation was proportional to the transplantation time and the number of transplanted cells. The earlier the transplantation, the larger the number of transplanted cells, the more significant recovery. These results suggest that bone marrow transplantation can effectively protect mice from death even after a significant secondary increase in plasma Hp after lethal dose irradiation (5 days after irradiation). In addition, the basic physiological indexes such as body weight and hemogram were recovered well. This cannot be realized by the existing biological dosimeter and Hp in serum plays an important role in the clinical treatment of severe patients with acute radiation injury.

In addition, current detection of indicators of hematopoietic recovery in patients with severe acute radiation injury (SARI) after HSCT treatment also depends on the recovery of absolute lymphocyte number and bone marrow cell [57, 58]. Our study showed that although the transplantation of exogenous bone marrow cells stimulated the increase of plasma Hp in mice in a short time (Fig. 4C), but at the later stage, it could promote the recovery of ARS in mice and inhibit plasma Hp. Transplantation with different number of bone marrow nucleated cells at 1, 3 and 5 days after lethal dose irradiation could protect mice from death and significantly inhibit the secondary rise of plasma Hp. The absolute number of lymphocytes, a main index of hematopoietic recovery in bone marrow transplantation, was significantly lagged behind the change of Hp. The number of lymphocytes in mice transplanted 1 day after irradiation did not recover until 20 days after irradiation, and the recovery of lymphocytes in other groups did not begin until 25 days after irradiation. The plasma Hp concentration of mice transplanted 1 day after irradiation did not show a second increase; the plasma Hp concentrations of mice transplanted 3 days after irradiation showed a mild second increase, and decreased to normal 11 days after irradiation. Bone marrow stem cell transplantation, especially at 5 days after irradiation, can protect the mice from death and inhibited the persistent elevation of Hp, which decreased significantly at 14 days after irradiation. Analysis showed that the dynamic changes of plasma Hp before and after transplantation were similar to the time dynamics of lymphocyte recovery. These results indicate the change of plasma Hp is closely related to the post-transplantation status of mice, suggesting the recovery of plasma Hp can be used as a directly indicator of post-transplantation healing of mice. Although the number of lymphocytes recovered earlier, it recovered slowly and was not significant in clinical tests. It usually reached the clinical safety index at 20 days after irradiation. Therefore, the decrease in plasma Hp after transplantation reflects a more sensitive and effective indicator of the success and recovery of bone marrow transplantation.

In this study, we found plasma Hp was elevated dose-dependently in mice at the early stage of radiation injury and patients after radiotherapy, suggesting that plasma Hp levels can be an early marker of radiation injury. Moreover, we detected a secondary elevation of plasma Hp in mice on the 3rd to 5th day after fatal irradiation, and bone marrow transplantation on the 3rd to 5th day after fatal irradiation effectively saved the mice from death, suggesting that monitoring the dynamic changes in plasma Hp concentrations after irradiation is helpful for the diagnosis of fatal radiation injury. This study completed relevant experiments to support the notion of the plasma Hp as a marker molecule of radiation injury in a mouse model, which provided insightful research and basis for subsequent validation experiments in human.

## Conclusions

Our results provide the first evidence that plasma Hp concentration can be used not only as an early bio-dosimeter of ionizing radiation, but also as a biomarker of fatal radiation injury. The second increase in plasma Hp after acute irradiation can be an important indicator of patients who should undergo HSCT, patients in urgent need of transplantation can be treated in a higher priority when the donor is limited. And the decrease in plasma Hp after transplantation is an indicator to verify the post-recovery status. Its role in the triage of injuries and early recognition and treatment of the severely wounded after nuclear accidents can be explored perspectively.

## Supplementary Information

**Additional file 1.** Supplemental materials： Figure S1–S5 and Table S1–S4.

## Data Availability

The datasets used and/or analyzed during the current study are available from the corresponding author on reasonable request.

## Abbreviations

TBI: Total body irradiation
PBI: Partial body irradiation
Hp: Haptoglobin
ARS: Acute radiation syndrome
BMT: Bone marrow transplantation
ELISA: Enzyme linked immunosorbent assay
HSCT: Hematopoietic stem cell transplantation
