# Supplementary material for: Haptoglobin is an early indicator of survival after radiation-induced severe injury and bone marrow transplantation in mice
Source: Stem Cell Res Ther. 2022 Sep 6;13:461. doi: 10.1186/s13287-022-03162-x (PMC9450283; doi:10.1186/s13287-022-03162-x)
Supplement: Supplementary file 1 — Additional file 1. Supplemental materials： Figure S1–S5 and Table S1–S4. [file 13287_2022_3162_MOESM1_ESM.docx]

**Figure S1. Weight and hemogram changes in mice after exposure to different doses of TBI.**

Weight, White blood cell count, Hemoglobin and Platelet count. Data are mean ± SD of n = 8 mice per group, statistical significance by Student’s t-test. ***, P<0.001. *, P<0.05. Hp, haptoglobin; TBI, total body irradiation.

**Table S1. Fold change and P values associated with two-tailed Student’s t-test for Hp concentrations after comparing control and irradiated groups.** Hp, haptoglobin.

**Figure S2. Expression of Hp mRNA in TBI mice and hemogram changes of mice after PBI.**

(A)Expression of Hp mRNA in normal mice and irradiated mice. (B) White blood cell count, Hemoglobin, Platelet count. Data are mean ± SD of n = 8 mice per group, statistical significance by Student’s t-test. *, P<0.05, and **, P<0.01 in the TBI irradiated mice compared with the control mice. Hp, haptoglobin; PBI, partial body irradiation; TBI, total body irradiation.

**Table S2. Clinical parameters and corresponding serum Hp concentration in 18 NPC patients.** NPC, nasopharyngeal cancer; RT, radiation; Hp, haptoglobin. The mean concentration of Hp after radiotherapy(4108μg/ml) was significantly higher than that before treatment (2015μg/ml), P=0.0023.

**Figure S3. Expression changes of Hp in mice and hemogram changes of mice after exposure and treatment with A.**

(A) White blood cell count, Hemoglobin, and Platelet count in irradiated mice (B)Hp was measured using ELISA in 0Gy, 10Gy+A, irradiated female C57BL/6J mice at -3, 1, 2, 3, 5, 7, 11 and 14 days post-irradiation. Data are mean ± SD of n = 8 mice per group, statistical significance by Student’s t-test.**, P<0.01, and ***, P<0.001 in the irradiated mice compared with the control mice. A, amifostine; Hp, haptoglobin.

**Table S3. The sublethal versus lethal Hp level predicts effect of the radioprotective agents.** Hp, haptoglobin.

**Figure S4.** The bone marrow injury was observed in mice after 6.5Gy irradiation.

(A) HE staining was performed in mouse femoral bone marrow and (B) Bone marrow nucleated cell has been counted. Data are mean ± SD of n = 3 mice per group, statistical significance by Student’s t-test. *, P<0.05; **, P<0.01; ****, P<0.0001.

**Figure S5. Weight and hemogram changes of bone marrow transplantation in mice with severe radiation injury.**

(A) Weight, White blood cell count, Hemoglobin and Platelet count. Data are mean ± SD of n = 6 mice per group except 10 Gy-3d-T-2(n=5), statistical significance by Student’s t-test. *, P<0.05; **, P<0.01; ***, P<0.001. Hp, haptoglobin; TBI, total body irradiation.

**Table S4. P values associated with two-tailed Student’s t-test for Hp concentrations after comparing with the previous time point.** Hp, haptoglobin.

Figure S1. Weight and hemogram changes in mice after exposure to different doses of TBI.

Weight

White blood cell count

Platelet count

Hemoglobin

Figure S2. Expression of Hp mRNA in TBI mice and hemogram changes of mice after PBI.

A

B

Platelet count

Hemoglobin

White blood cell count

Figure S3. Expression changes of Hp in mice and hemogram changes of mice after exposure.

Hemoglobin

White blood cell count

A

Platelet count

B

Figure S4. The bone marrow injury was observed in mice after 6.5Gy irradiation.
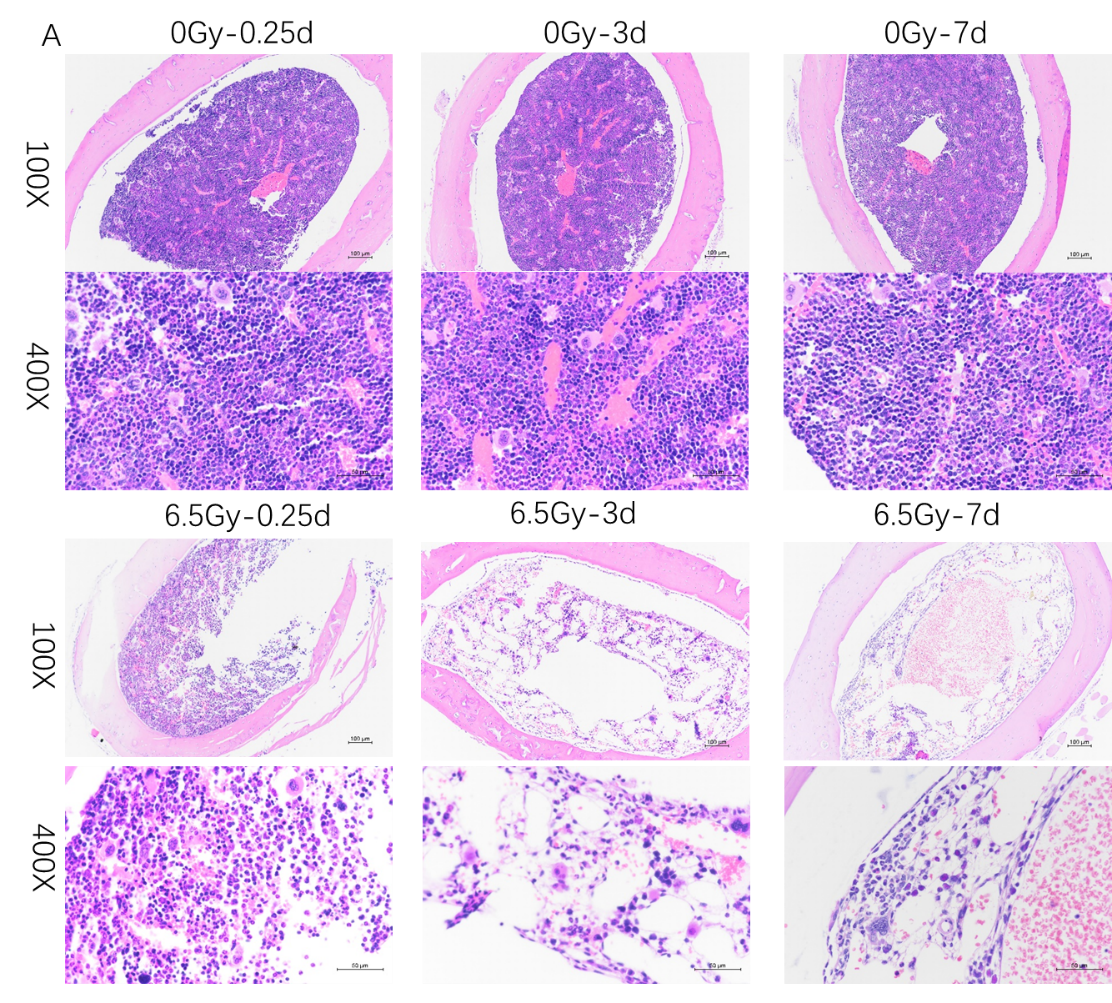


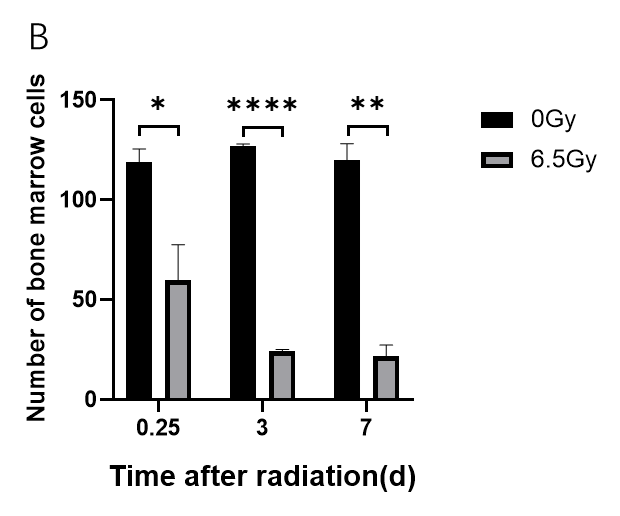


Figure S5. Weight and hemogram changes of bone marrow transplantation in mice with severe radiation injury.

White blood cell count

Weight

Platelet count

Hemoglobin

Table S1. Fold change and P values associated with two-tailed Student’s t-test for Hp concentrations after comparing control and irradiated groups.

| Comparison  Fold change  and *P* value | Time post-irradiation (days) | | | | | | | | | |
| --- | --- | --- | --- | --- | --- | --- | --- | --- | --- | --- |
|  | 0.125 | 0.25 | 0.5 | 1 | 2 | 3 | 5 | 7 | 11 | 14 |
| 0.2Gy vs. Control | 0.71  0.4762 | 0.22  0.1994 | 1.00  0.8739 | 4.91  0.3895 | 10.18  0.1446 | 10.93  0.1699 | 1.75  0.4622 | 0.87  0.3553 | 0.22  0.3009 | 0.51  0.3489 |
| 0.5Gy vs. Control | 0.77  0.2968 | 0.38  0.2354 | 1.00  0.8766 | 2.55  0.3073 | 7.27  0.1638 | 5.58  0.6807 | 2.07  0.3630 | 0.48  0.1043 | 0.33  0.4197 | 0.25  0.8149 |
| 1Gy vs. Control | 0.54  0.9998 | 0.28  0.2865 | 3.16  0.0008 | 11.85  0.0048 | 13.32  0.0862 | 3.64  0.8031 | 1.05  0.9041 | 0.41  0.0824 | 2.47  0.5197 | 0.61  0.3856 |
| 2Gy vs. Control | 0.51  0.8968 | 0.79  0.0591 | 8.67  <0.0001 | 20.18  <0.0001 | 25.11  0.0061 | 14.87  0.0847 | 1.00  0.9418 | 0.13  0.0261 | 0.12  0.2269 | 4.72  0.4196 |
| 4Gy vs. Control | 1.09  0.0622 | 1.62  <0.0001 | 16.22  <0.0001 | 38.19  <0.0001 | 31.58  0.0001 | 26.37  0.0002 | 1.02  0.9142 | 0.20  0.0325 | 0.12  0.2263 | 0.10  0.0194 |
| 6Gy vs. Control | 1.75  0.2772 | 2.78  0.0021 | 23.34  <0.0001 | 48.44  <0.0001 | 40.34  <0.0001 | 30.47  0.0004 | 41.48  0.0034 | 19.84  0.0928 | 0.26  0.3444 | 4.15  0.4167 |
| 8Gy vs. Control | 2.21  0.0255 | 3.27  0.0006 | 24.41  <0.0001 | 50.72  <0.0001 | 46.61  0.0002 | 38.98  0.0102 | 61.29  0.0091 | 184.83  0.0109 | 261.62  0.0051 | 112.19 |
| 10Gy vs. Control | 2.43  0.0308 | 4.15  0.0001 | 26.58  <0.0001 | 54.14  <0.0001 | 43.93  <0.0001 | 71.98  <0.0001 | 79.84  <0.0001 | 286.20  <0.0001 |  |  |

Table S2. Clinical parameters and corresponding serum Hp concentration in 18 NPC patients.

| Patient  No. | Sex/Age  (year) | Stage | | Hp concentration(μg / ml) | | Tumor type |
| --- | --- | --- | --- | --- | --- | --- |
|  |  | Overall | TNM | Before RT | After RT |  |
| 1 | M/51 | Ⅲ | T3N2M0 | 756 | 1633 | NPC |
| 2 | M/47 | Ⅲ | T3N2M0 | 888 | 2595 | NPC |
| 3 | M/53 | Ⅲ | T3N2M0 | 4042 | 3536 | NPC |
| 4 | M/57 | Ⅲ | T3N2M0 | 1273 | 2371 | NPC |
| 5 | F/56 | ⅣA | T2N3M0 | 1419 | 2129 | NPC |
| 6 | F/57 | Ⅲ | T3N1M0 | 3046 | 2754 | NPC |
| 7 | M/63 | Ⅲ | T3N2M0 | 4286 | 4468 | NPC |
| 8 | F/56 | Ⅲ | T2N2M0 | 1042 | 4736 | NPC |
| 9 | F/67 | Ⅲ | T2N2M0 | 1554 | 2112 | NPC |
| 10 | F/34 | Ⅲ | T3N2M0 | 820 | 2958 | NPC |
| 11 | F/60 | Ⅲ | T3N2M0 | 1106 | 9837 | NPC |
| 12 | F/48 | Ⅲ | T3N2M0 | 1072 | 2958 | NPC |
| 13 | F/40 | Ⅲ | T2N2M0 | 421 | 1389 | NPC |
| 14 | F/60 | Ⅳ | T4N2MX | 3229 | 5585 | NPC |
| 15 | F/61 | Ⅲ | T2N2M0 | 646 | 7025 | NPC |
| 16 | M/70 | ⅣA | T4N1M0 | 4567 | 9844 | NPC |
| 17 | M/67 | ⅣB | T4N2M1 | 4542 | 4511 | NPC |
| 18 | M/61 | ⅣB | T4N2M1 | 1570 | 3494 | NPC |

Table S3. The sublethal versus lethal Hp level predicts effect of the radioprotective agents.

| Group | No. |  | Hp concentration (μg/ml) | | | | | |  | Date of death  (day) |
| --- | --- | --- | --- | --- | --- | --- | --- | --- | --- | --- |
|  |  | Day -3 | Day 1 | Day 2 | Day 3 | Day 5 | Day 7 | Day11 | Day14 |  |
| 10Gy | 1824 | 2.0 | 904.3 | 468.3 | 675.8 | 1435.9 | 5272.6 |  |  | 11 |
|  | 1823 | 20.0 | 1244.8 | 562.2 | 776.3 | 459.9 |  |  |  | 6 |
|  | 1822 | 11.2 | 1004.2 | 440.6 | 826.1 | 1115.6 | 4306.2 |  |  | 11 |
|  | 1821 | 8.7 | 1198.6 | 691.2 | 926.6 | 1101.1 | 5395.6 |  |  | 11 |
|  | 1820 | 12.6 | 1277.0 | 391.7 | 763.8 | 879.6 | 4174.4 |  |  | 9 |
|  | 1819 | 13.2 | 1608.6 | 405.6 | 848.6 | 768.6 | 2343.7 | 7550.4 |  | 12 |
|  | 1818 | 16.0 | 1103.9 | 525.8 | 959.2 | 1386.5 | 4877.1 |  |  | 10 |
|  | 1817 | 3.7 | 1036.2 | 888.2 | 685.8 | 1431.5 | 1661.1 |  |  | 10 |
| 10Gy+amifostine | 1832 | 42.4 | 1157.6 | 545.9 | 264.1 | 368.4 | 70.1 | 1.5 | 2.5 | - |
|  | 1831 | 23.2 | 1387.5 | 473.2 | 212.7 | 319.5 | 116.1 | 3.6 | 1.6 | - |
|  | 1830 | 2.1 | 1287.6 | 363.0 | 176.5 | 1046.0 | 647.3 | 2.1 | 1.6 | - |
|  | 1829 | 19.7 | 1628.3 | 553.4 | 256.5 | 966.2 | 219.9 | 2.1 | 3.2 | - |
|  | 1828 | 5.2 | 1259.5 | 583.5 | 856.5 | 1663.7 | 2006.4 | 6970.7 |  | 12 |
|  | 1827 | 19.4 | 1813.6 | 473.2 | 275.4 | 430.9 | 19.5 | 2.1 | 1.9 | - |
|  | 1826 | 13.3 | 1380.5 | 637.4 | 346.7 | 777.1 | 443.3 | 2.2 | 2.2 | - |
|  | 1825 | 17.2 | 1287.6 | 718.8 | 232.8 | 481.1 | 306.5 | 2.9 | 2.7 | - |

Table S4. P values associated with two-tailed Student’s t-test for Hp concentrations after comparing with the previous time point.

| *P* value | Time post-irradiation (days) | | | | | | |
| --- | --- | --- | --- | --- | --- | --- | --- |
|  | 1 | 2 | 3 | 5 | 7 | 11 | 14 |
| 10 Gy | <0.0001 | 0.0017 | 0.0510 | 0.0213 | 0.0049 | 0.0003 |  |
| 10 Gy-1d-T | <0.0001 | 0.0002 | 0.1208 | 0.0004 | 0.9576 | 0.2791 | 0.9288 |
| 10 Gy-3d-T-1 | 0.0004 | 0.2807 | 0.2745 | 0.0371 | 0.0039 | 0.0319 | 0.7891 |
| 10 Gy-3d-T-2 | 0.0001 | 0.8506 | 0.3106 | 0.0580 | 0.3098 | 0.1077 | 0.8856 |
| 10 Gy-5d-T-1 | 0.0002 | 0.4582 | 0.3712 | 0.0041 | <0.0001 | 0.4149 | 0.0026 |
| 10 Gy-5d-T-2 | <0.0001 | 0.0616 | 0.8293 | 0.0104 | 0.0178 | 0.1562 | 0.0104 |
| 10 Gy-5d-T-3 | <0.0001 | 0.4669 | 0.0633 | 0.0443 | 0.0054 | 0.2549 | 0.0243 |
